# Supplementary material for: Evaluating the effectiveness of a group-based resilience intervention versus psychoeducation for emergency responders in England: A randomised controlled trial
Source: PLoS One. 2020 Nov 12;15(11):e0241704. doi: 10.1371/journal.pone.0241704 (PMC7660584; doi:10.1371/journal.pone.0241704)
Supplement: S2 File — (PDF) [file pone.0241704.s002.pdf]

Evaluating the effectiveness of a group-based resilience intervention versus psychoeducation for emergency responders in England: A randomised controlled trial

Short Title: IMPROVE RESILIENCE
Ethics Ref: MS-IDREC-C1-2015-059
ISRCTN: ISRCTN79407277

**Principal Investigator:**	Dr Jennifer Wild, Department of Experimental Psychology, University of Oxford
**Research Staff:**	Shama El-Salahi, Department of Experimental Psychology, University of Oxford<br><br>Michelle Degli Esposti, Department of Experimental Psychology, University of Oxford
**Funder:**	Mind

Confidentiality Statement
This document contains confidential information that must not be disclosed to anyone other than the Investigator Team, host organisation, and members of the Research Ethics Committee, unless authorised to do so.

TABLE OF CONTENTS

Chapter	Title	Page
1	Key Trial Contacts	3
2	Synopsis	4
3	Abstract	6
4	Background and Rationale	6
5	Objectives and Outcome Measures	7
6	Trial Design	8
7	Participant Identification	9
8	Trial Procedures	9
9	Interventions	10
10	Safety Reporting	11
11	Statistics	14
12	Data Management	15
13	Quality Assurance Procedures	15
14	Ethical and Regulatory Considerations	15
15	Finance and Insurance	16
16	Publication Policy	16
17	References	16
18	Appendix A: Trial Flow Chart	19
19	Appendix B: Amendment History	20

1. KEY TRIAL CONTACTS

**Principal Investigator**	Dr Jennifer Wild<br>Centre for Anxiety Disorders and Trauma<br>Department of Experimental Psychology<br>University of Oxford<br>The Old Rectory<br>Paradise Square<br>Oxford OX1 1TW<br>Email: jennifer.wild@psy.ox.ac.uk<br>TEL: 01865 618612
**Research Staff**	Shama El-Salahi, Department of Experimental Psychology, University of Oxford<br>Shama.elsalahi@psy.ox.ac.uk<br>TEL: 01865-618-600<br><br>Michelle Degli Esposti, Department of Experimental Psychology, University of Oxford<br>Michelle.degliesposti@psy.ox.ac.uk<br>TEL: 01865-618-600
**Statistician**	Dr Graham Thew<br>Department of Experimental Psychology<br>University of Oxford<br>The Old Rectory<br>Paradise Square<br>Oxford OX1 1TW<br>Email: esther.beierl@psy.ox.ac.uk<br>Tel: 01865 618600

2. **SYNOPSIS**

Trial Title	Evaluating the effectiveness of a group-based resilience intervention versus psychoeducation for emergency responders in England: A randomised controlled trial	
Internal ref. no. (or short title)	IMPROVE RESILIENCE	
Trial Design	Randomized controlled trial	
Trial Participants	Emergency workers (police, paramedics, firefighters, search and rescue personnel) in England	
Planned Sample Size	430	
Training duration	6 weeks	
Follow up duration	3 months	
Planned Trial Period	12 May 2015 – 31 March 2016	
	Objectives	Outcome Measures
Primary	**1**. Is Mind's group-based resilience intervention more effective than accessing already available psychoeducation about mental health (online psychoeducation) for improving wellbeing, resilience, general self-efficacy, social capital, use of social support, confidence to manage mental health and in reducing days off work due to illness?	**1. Wellbeing:** The Warwick Edinburgh Mental Wellbeing scale [1].<br>2. **Resilience:** The Connor-Davidson Resilience Scale [2]<br>3. **General Self-Efficacy**: The General Self-Efficacy Scale [3]<br>4. **Social Capital:** the Social Participation scale [4]<br>5. **Social Support scale** adapted from Sarason et al's scale [5], which has two subscales, Social Support (Home) and Social Support (Work).<br>6. **Confidence to manage mental health:** A one-item question to assess the degree to which participants feel confident to manage their mental health<br>7. **Days off due to illness:** An unpublished questionnaire to assess days off due to illness.
Secondary	**1.** Does Mind's group-based resilience intervention lead to greater improvement in secondary outcome measures (depressive attributions,	**1.**<br>**Depressive attributions:** The Attributions Questionnaire [6]

	coping, maladaptive responses to intrusive memories, rumination, depression, anxiety, problematic alcohol use, problem solving) than psychoeducation?	**Coping**:<br>Adaptive Coping: Brief Coping Behaviour Questionnaire [7] (3 subscales: active coping, use of emotional support, and acceptance)<br>Dysfunctional Coping: Brief Coping Behaviour Questionnaire [7] (5 subscales: self-distraction, denial, substance use, self-blame, behavioural disengagement) and wishful thinking subscale [8].<br><br>**Maladaptive responses to intrusive memories:** Responses to Intrusions Questionnaire [8]<br><br>**Rumination:** The Ruminative Responses Scale [9]<br><br>**Trauma & PTSD:** trauma screener, adapted from the Clinician Administered PTSD Scale for DSM-5 (CAPS-5) [10]<br>The PTSD Checklist for DSM-5 [11]<br><br>**Depression** Patient Health Questionnaire-9 [12]<br><br>**Anxiety** General Anxiety Disorder 7 (GAD-7) [13]<br><br>**Problematic alcohol use** The Alcohol Use Disorders Identification Test [14]<br><br>**Problem solving** unpublished questionnaire used in previous evaluations of Mind's resilience intervention. [15]
	**2.** Does neuroticism predict the degree of change participants experience in wellbeing, resilience, self-efficacy and social capital as a result of the intervention?	**2.**<br>**Neuroticism** neuroticism subscale of the Short-Form Revised Eysenck Personality Questionnaire [16]
Treatment conditions	1. Group-based resilience intervention<br>2. Psychoeducation	

3. Abstract

Introduction: Emergency workers dedicate their lives to promoting health and public safety yet experience higher rates of mental ill health compared to the general population. Effective interventions to improve their resilience and wellbeing are urgently needed.

Design, Methods and Analysis: We will conduct a multicentre, parallel-group, randomised controlled trial. A total of N=430 emergency workers will be recruited and randomly allocated to the interventions (group-based resilience or psychoeducation) on a 3:1 ratio. Follow-up will occur after the interventions and at 12 weeks. We hypothesise that the group-based resilience intervention will be more effective than psychoeducation in improving resilience, wellbeing, self-efficacy, and social capital, as well as in improving emergency workers' confidence to manage their mental health and reduce days off work due to illness. We hypothesise that neuroticism will predict the degree of change participants will experience in wellbeing, resilience, self-efficacy and social capital.

Ethics and dissemination: The Medical Sciences Inter-Divisional Research Ethics Committee granted ethical approval on 1 April, 2015 at the University of Oxford, valid until 31 March, 2016. Reference: MS-IDREC-C1-2015-059.

4. BACKGROUND AND RATIONALE

Resilience is what determines how people react to adversity, how it affects the outcomes of their lives. Resilience can be trained and with treatment, people can become more resilient [i.e., 2]. Research suggests that resilient people are less likely to suffer from mental health problems [17].

Mind has developed a model of resilience and an intervention based on this model to improve the mental health resilience of at risk groups. The intervention has already demonstrated promising effects for pregnant women and new mothers at risk of social isolation, and unemployed men.

Another group at risk of developing mental health problems are emergency service workers who experience daily stressors and witness frequent trauma as part of their job. They dedicate their lives to improving public health yet suffer higher rates of mental ill health compared to the general population. Can Mind's resilience intervention help this group?

Mind's model of resilience builds on the five ways to wellbeing, a set of evidence-based public mental health messages, identified by the New Economics Foundation, aimed at improving the mental health and wellbeing of the whole population.

Mind's resilience programme contributes towards the achievement of Mind's visionary Unstoppable Together strategy (2012–2016), which includes supporting people who are at risk of mental health problems to build resilience and to stay well. A key aim of Mind's resilience intervention is to improve wellbeing. This is important as wellbeing predicts a broad range of general health outcomes including, for example, working days lost through illness five years later [18], likelihood of stroke six years later and of cardio-vascular disease ten years later [19].

The intervention also aims to improve social capital, the main aspects of which include fostering a sense of belonging in neighbourhoods and communities, and accessing social networks and support. Research has shown that higher levels of social capital are linked to better health, higher educational achievement, better employment outcomes, and lower crime rates (Office for National Statistics).

Finally, Mind's resilience intervention aims to develop psychological coping strategies drawn from stress management and mindfulness interventions, an aspect that is of particular relevance to populations with high risk of exposure to stressful and potentially traumatic events. In a seminal study of ambulance workers, Clohessy and Ehlers (1999) demonstrated that particular psychological coping strategies were linked to lower levels of mental ill health. Shepherd and Wild (2013) demonstrated that particular types of thoughts following stressors were linked with better coping in paramedics [20].

A rigorous evaluation is essential to determine the effectiveness of the intervention.

5. OBJECTIVES AND OUTCOME MEASURES

**Objectives**	**Outcome Measures**	**Timepoint(s) of evaluation of this outcome measure (if applicable)**
**Primary Objectives**<br><br>**1**. Is Mind's group-based resilience intervention more effective than accessing already available psychoeducation about mental health (online psychoeducation) for improving wellbeing, resilience, general self-efficacy, social capital, use of social support, confidence to manage mental health and in reducing days off work due to illness?	1. **Wellbeing:** The Warwick Edinburgh Mental Wellbeing scale [1].<br>2. **Resilience:** The Connor-Davidson Resilience Scale [2]<br>3. **General Self-Efficacy**: The General Self-Efficacy Scale [3]<br>4. **Social Capital:** the Social Participation scale [4]<br>5. **Social Support scale** adapted from Sarason et al's scale [5], which has two subscales, Social Support (Home) and Social Support (Work).<br>6. **Confidence to manage mental health:** A one-item question to assess the degree to which participants feel confident to manage their mental health<br>7. **Days off due to illness:** An unpublished questionnaire to assess days off due to illness.	1.<br>Baseline<br>6 weeks<br>12 weeks
**Secondary Objectives**<br><br>**1.** Does Mind's group-based resilience intervention lead to greater improvement in secondary outcome measures (depressive attributions, dysfunctional coping, maladaptive	**1. Depressive attributions:** The Attributions Questionnaire [6]<br><br>**Coping**:<br>Adaptive Coping: Brief Coping Behaviour Questionnaire [7] (3	1.<br>Baseline<br>6 weeks<br>12 weeks

responses to intrusive memories, rumination, depression, anxiety, problematic alcohol use, problem solving) than psychoeducation?	subscales: active coping, use of emotional support, and acceptance)<br>Dysfunctional Coping: Brief Coping Behaviour Questionnaire [7] (5 subscales: self-distraction, denial, substance use, self-blame, behavioural disengagement) and wishful thinking subscale [8].	
**2.** Does neuroticism predict the degree of change participants experience in wellbeing, resilience, self-efficacy and social capital as a result of the intervention?	**Maladaptive responses to intrusive memories:** Responses to Intrusions Questionnaire [8]<br><br>**Rumination:** The Ruminative Responses Scale [9]<br><br>**Trauma & PTSD:** trauma screener, adapted from the Clinician Administered PTSD Scale for DSM-5 (CAPS-5) [10]<br>The PTSD Checklist for DSM-5 [11]<br><br>**Depression** Patient Health Questionnaire-9 [12]<br><br>**Anxiety** General Anxiety Disorder 7 (GAD-7) [13]<br><br>**Problematic alcohol use** The Alcohol Use Disorders Identification Test [14]<br><br>**Problem solving** unpublished questionnaire used in previous evaluations of Mind's resilience intervention. [15]	
	**2.**<br>**Neuroticism** neuroticism subscale of the Short-Form Revised Eysenck Personality Questionnaire [16]	2. Baseline

6. TRIAL DESIGN

The design is a parallel group randomised controlled trial comparing a group-based resilience intervention to psychoeducation about mental health.

7. PARTICIPANT IDENTIFICATION

7.1. Trial Participants

Participants will be emergency service workers (police, paramedics, firefighters, search and rescue personnel) not suffering from PTSD or major depression.

7.1.1 Inclusion Criteria

- Aged 18 and above.
- Police officers, paramedics, firefighters, or search and rescue personnel
- Access to internet.
- Willing to be randomly allocated.

7.1.2. Exclusion Criteria

- Current symptoms of PTSD or major depression requiring treatment

8. TRIAL PROCEDURES

8.1 Recruitment

Recruitment will be conducted in collaboration with local Mind centres and local emergency services at nine selected sites across England: Andover, Brighton and Hove, Coastal West Sussex, Dudley, Southampton, Birmingham, Oxfordshire, Cambridgeshire, and Peterborough and Fenland. The Principal Investigator or Mind staff will give talks at emergency service sites, circulate emails about the course, put up posters at emergency services, provide leaflets about Mind's Blue Light Programme which references the course, and post on social media.

Emergency workers will be directed to Mind's website where they can sign up for the trial via a link to the registration survey on Qualtrics, a secure online software platform. Participants can read and print a PDF copy of the Participant Information Sheet and pause the registration process to discuss questions with the research assistant over the telephone. If they decide to take part, they will be emailed an individualised link where they can log-in, re-read the Participant Information Sheet, and complete a consent form and two short screening questionnaires.

8.2 Screening and Eligibility Assessment

Participants will be screened for depression and suicidal ideation using the Patient Health Questionnaire 9 (PHQ-9) [12], and for post-traumatic stress using the Post-Traumatic Stress Disorder Checklist for DSM-5 (PCL-5) [11]. They will be considered eligible if they score below 10 on the PHQ-9, below 33 on the PCL-5, and 0 on question nine of the PHQ-9, which assesses suicidal ideation. If participants score above these cut-off points, they will have a telephone call with the researchers to discuss whether their symptoms interfered with their lives and whether they wish treatment. They will be re-included in the study if their symptoms have little impact on their lives and they do not wish treatment, otherwise they will be excluded and signposted for evidence-based psychological treatment within local Improving

Access to Psychological Therapies services. To reduce the risk of participants dropping out, eligible participants will be asked to confirm they can commit to a six-week programme before they are randomised.

Eligible participants who wish to participate and have given consent will be emailed a link to complete their initial baseline questionnaires.

8.3 Informed Consent

After receiving detailed written and verbal information about the exact nature of the trial and what it will involve for them, and after having had an opportunity to ask questions, if agreeing to participate in the trial, the participant will indicate that they give consent by ticking a box on the electronic consent form.

The information sheet and consent form state the participant is free to withdraw from the trial at any time for any reason without penalty, by advising the researchers of this decision.

The participant will be allowed as much time as wished to consider the information, and the opportunity to question the Investigator or other independent parties to decide whether they will participate in the trial. Electronic informed consent will be obtained prior to completing the two screening questionnaires.

8.4 Randomisation

Random allocation to the two trial conditions (Resilience Group, Psychoeducation) will be on a 3:1 ratio, stratified by site and gender using a programme called Minim, which the research assistant will co-ordinate.

8.5 Subsequent Assessments

Participants will complete questionnaires at baseline and follow-up questionnaires at 6 and 12 weeks.

8.6 Discontinuation/Withdrawal of Participants from Trial Treatment

Each participant has the right to withdraw from the trial at any time. The reason for withdrawal will be recorded.

8.7 Definition of End of Trial

The end of trial is the date of the last follow up with the last participant.

9. INTERVENTIONS

The resilience intervention is a six-week, group-based course developed for Mind by Shaun Goodwin, a psychotherapist with expertise in transpersonal counselling, and previously delivered in their work with new mothers and men at risk of social isolation (Robinson et al.,

2014) [15]. The intervention includes information about mental health and experiential exercises drawn from stress management and mindfulness, with the overarching aim to improve wellbeing and use of adaptive coping strategies, such as social support. Table 1 shows an overview of the weekly content. Homework exercises are set between each session to reinforce learning. Each group session lasts 2.5 hours. Mind facilitators attend a one-day workshop on how to deliver the intervention and then weekly supervision whilst it is ongoing.

Table 1. Overview of the Weekly Content of the Resilience Intervention

Session	Content
1	Hopes and Expectations. Looking at how stress affects thoughts, feelings, physical wellbeing and behaviour.
2	Understanding anxiety and learning why we react the way we do. Identifying distorted thoughts and moods.
3	How we can limit ourselves through habitual negative thoughts and moods. Challenging distorted negative thoughts and moods.
4	Managing worry. Managing stress. 'Time for me' and learning how to relax and the importance of doing so. Breathing techniques. Controlling panic.
5	Setting goals and challenges. Understanding passive anger and resistance. Learning about comfort zones and panic zones.
6	Reviewing learning. Planning for the future.
Throughout the course	A different relaxation technique is introduced in each session, including techniques based on mindfulness.

9.1 Online Psychoeducation

The comparison intervention includes psychoeducation about six topics that have been selected from a range freely available online from Mind's website https://www.mind.org.uk/information-support/types-of-mental-health-problems/, which the researchers have tailored for emergency workers. Each topic is delivered as an online module, one released each week for six weeks during the same six-week period that the resilience intervention takes place. Participants complete the modules remotely at a time during the week that suited them.

- Dealing with Stress
- Sleep Problems
- Anger
- Depression
- Post-traumatic Stress Disorder
- Mindfulness

9.2 Treatment fidelity

Each group session will be recorded using SanDisk MP3 players and the researchers will rate 10% of the sessions for adherence to protocol, using a short questionnaire that relates to the core elements for each of the six sessions.

10. SAFETY REPORTING

10.1 Definitions

Term	Definition
**Adverse Event (AE)**	Any untoward medical occurrence in a participant or clinical trial participant and which are not necessarily caused by or related to that product
**Serious Adverse Event (SAE)**	Any adverse event that -<br>• Results in death<br>• Is life-threatening*<br>• Required hospitalisation or prolongation of existing hospitalisation**<br>• Results in persistent or significant disability or incapacity<br>• Consists of a congenital anomaly or birth defect<br>• Other medically important condition***

***Note:** The term 'life-threatening' in the definition of serious refers to an event in which the trial participant was at risk of death at the time of the event or it is suspected that used or continued used of the product would result in the subject's death; it does not refer to an event which hypothetically might have caused death if it were more severe.

10.2 Causality

The Principal Investigator will assess each SAE to determine the causal relationship:

Relationship	Description	Reasonable possibility that the SAE may have been caused by the intervention?
**Unrelated**	There is no evidence of any causal relationship with the trial/intervention	No
**Unlikely**	There is little evidence to suggest there is a causal relationship with the trial/intervention (e.g. the event did not occur within a reasonable time after the intervention). There is another reasonable explanation for the event (e.g. the participant's clinical condition, other concomitant treatment).	No

**Possible**	There is some evidence to suggest a causal relationship with the trial/intervention (e.g. because the event occurs within a reasonable time after the intervention). However, the influence of other factors may have contributed to the event (e.g. participant's physical health or exposure to critical incident at work).	Yes
**Probable**	There is evidence to suggest a causal relationship and the influence of other factors is unlikely.	Yes
**Definite**	There is clear evidence to suggest a causal relationship and other possible contributing factors can be ruled out.	Yes

10.3. Expectedness

The Chief Investigator will assess each SAE to perform the assessment of expectedness. The expectedness assessment should be made according to the information as detailed in the protocol.

Reference Safety Information (RSI)

There are no expected side effects linked to either intervention.

Expectedness decisions must not take into account factors such as the participant population and participant history. Expectedness is not related to what is an anticipated event within a particular disease. SAEs which add significant information on specificity or severity of a known, already documented adverse event constitute unexpected events.

10.4 Procedures for Recording Adverse Events

All AEs occurring during the trial that are observed by the research team or reported by the participant, will be recorded. The following information will be recorded: description, date of onset and end date, severity, assessment of relatedness to intervention, and action taken. Follow-up information should be provided as necessary. The severity of events will be assessed on the following scale: 1 = mild, 2 = moderate, 3 = severe.

10.5 Reporting Procedures for Serious Adverse Events

All SAEs must be reported on the SAE reporting form to the REC within 15 working days of the Principal Investigator becoming aware of the event, using the Health Research Authority safety report form for a non-Clinical Trial of an Investigation of a Medicinal Product (non-CTIMP).
It will also be reviewed at the next Trial Steering Committee meeting. All SAE information must be recorded on an SAE form and faxed, or scanned and emailed, to the REC. Additional and further requested information (follow-up or corrections to the original case) will be detailed on a new SAE Report Form and faxed/emailed to the REC.

10.6 Monitoring for negative effects of treatment delivery over the internet

We will assess participant's evaluation of the internet modules and design through a feedback and rating form at the end of each module and in comments to the research assistant in emails or telephone calls.

11. STATISTICS

11.1. Description of Statistical Methods

11.1.1. Main Analysis

All analyses will be intent-to-treat. No interim analyses are planned. Linear mixed effect models will be used for the analysis of the primary and secondary outcome variables. Time (post-intervention, and three-month follow-up), treatment condition (resilience intervention or online psychoeducation [active control]), and the time-by-condition interaction will be entered as categorical fixed factors along with the stratification variables of gender and site. Baseline score will be included as a covariate, and a random effect of participant will be specified to account for between-person variation. When analysing secondary outcome measures, the baseline scores of the primary outcome measures will be included as additional covariates. All models will be estimated using restricted maximum likelihood estimation.

11.1.2 Secondary Analysis

A series of linear regressions will be performed to examine if baseline neuroticism scores predict the extent of pre-post change in wellbeing, resilience, self-efficacy, and social capital within the treatment group. Residualised gain scores will be used as the dependent variable in each analysis, and gender, site, and baseline score will be included as covariates.

11.2 The Number of Participants

We plan to recruit 430 participants.

Guidelines set by the Cabinet Office for this study suggested a target sample size of 430. We conducted a power analysis to confirm the sample would be large enough to detect an effect should one exist. We referred to a study by Kuehl et al. (2014) who compared a group-based 12-week stress management intervention for police officers against standard practice [21]. The intervention led to between-group improvements in wellbeing with small effect (d=0.34). Power was calculated with G*Power. In order to detect an effect of this size (i.e., effect size f=0.17) between two groups (three measurement points: pre-intervention, post-intervention, and follow-up), with 90% power at an alpha level of 0.05 requires a sample of N=318. Allowing for 20% attrition, a total sample size of N=398 would be required, suggesting that the target sample size was large enough to detect an effect.

11.3 The Level of Statistical Significance

Significance levels were set at $p < .05$.

11.4 Criteria for the Termination of the Trial

There are no stopping rules for the trial as it is a low risk non-CTIMP.

11.5 Inclusion in Analysis

Analyses will be intent-to-treat, i.e. all randomized participants will be included in the analysis.

12. DATA MANAGEMENT

12.1. Source Data

Source data will be captured online via Qualtrics software. Access to the system will be restricted to named study personnel only and via password protection. We have arranged with the University of Oxford IT services for the files to be encrypted and backed up on a weekly basis using the Tivoli Storage Manager, the data are copied to three separate tapes. One copy resides in the Tape Robot in the IT Services Machine room. The other two copies are held in locked fireproof safes, one onsite at IT Services, one offsite in locked premises. The data on the tapes are inaccessible without the TSM database. The data on the offsite tapes are encrypted.

12.2. Access to Data

Direct access will be granted to authorised representatives from the host institution and the regulatory authorities to permit trial-related monitoring, audits and inspections.

13. QUALITY ASSURANCE PROCEDURES

The trial will be conducted in accordance with the current approved protocol, relevant regulations and standard operating procedures. Regular monitoring will be performed. Data will be evaluated for compliance with the protocol, and completeness and accuracy in relation to source documents (e.g., for SCID-5). The trial steering committee will regularly view recruitment rates, compliance with delivery of different training programmes and completeness of data collection.

14. ETHICAL AND REGULATORY CONSIDERATIONS

14.1. Declaration of Helsinki

The Investigator will ensure that this trial is conducted in accordance with the principles of the Declaration of Helsinki.

14.2. Guidelines for Good Clinical Practice

The Investigator will ensure that this trial is conducted in accordance with relevant regulations and with Good Clinical Practice.

14.3. Approvals

The protocol, informed consent form, participant information sheet and advertising material has been submitted and approved by the University of Oxford Inter-Divisional Medical Sciences Research Ethics Committee (REC).

14.4. Participant Confidentiality

The trial staff will ensure that the participants' anonymity is maintained. The participants will be identified only by a participant ID number on all trial documents and any electronic database, with the exception of the signed consent form, where participant initials may be added. All documents will be stored securely and only accessible by trial staff and authorised personnel. The trial will comply with the Data Protection Act, which requires data to be anonymised as soon as it is practical to do so.

15. FINANCE AND INSURANCE

15.1. Funding

The trial is funded by Mind, awarded to Jennifer Wild.

15.2. Insurance

The University has a specialist insurance policy in place which would operate in the event of any participant suffering harm as a result of their involvement in the research (Newline Underwriting Management Ltd, at Lloyd's of London).

16. PUBLICATION POLICY

The results of the trial will be published in peer-reviewed international journals and will be made open access.

17. REFERENCES

1. Tennant R, Hiller L, Fishwick R, Platt S, Joseph S, Weich S, et al. The Warwick-Edinburgh Mental Well-Being Scale (WEBWBS): development and UK validation. *Health Qual Life Outcomes*. 2007;5: 63.

2. Connor KM, Davidson JRT. Development of a new resilience scale: The Connor-Davidson Resilience Scale (CD-RISC). *Depress Anxiety*. 2003;18: 76-82.

3. Schwarzer R, Jerusalem M. Generalized Self-Efficacy scale. In: Weinman J, Wright S, Johnston M, editors. *Measures in health psychology: A user's portfolio. Causal and control beliefs*. Windsor, UK: NFER-NELSON; 1995. pp. 35-37.

4. Taylor CT, Alden LE. To see ourselves as others see us: an experimental integration of the intra and interpersonal consequences of self-protection in social anxiety disorder. *J Abnorm Psychol*. 2011;120(1): 129-141.

5. Sarason IG, Sarason BR, Shearin EN, Pierce GR. A brief measure of social support: Practical and theoretical implications. *J Soc Pers Relat*. 1987;*4:* 497-510.

6. Kleim B, Gonzalo D, Ehlers A. The Depressive Attributions Questionnaire (DAQ): Development of a Short Self-Report Measure of Depressogenic Attributions. *J Psychopathol Behav Assess*. 2011;33(3): 375-385.

7. Carver CS, Scheier MF, Weintraub JK. Assessing coping strategies: A theoretically based approach. ***J Pers Soc Psychol.*** 1989;**56**(2): 267-283.

8. Clohessy S, Ehlers A. PTSD symptoms, response to intrusive memories and coping in ambulance service workers. *Br J Clin Psychol*. 1999;38(3): 251-265.

9. Treynor W, Gonzalez R, Nolen-Hoeksema. Rumination Reconsidered: A Psychometric Analysis. *Cognit Ther Res*. 2003;27(3): 247-259.

10. Weathers FW, Blake DD, Schnurr PP, Kaloupek DG, Marx BP, Keane TM. *The Clinician-Administered PTSD Scale for DSM-5 (CAPS-5)*. Interview available from the National Center for PTSD at www.ptsd.va.gov; 2013.

11. Weathers FW, Litz BT, Keane TM, Palmieri PA, Marx BP, Schnurr PP. *The PTSD Checklist for DSM-5 (PCL-5).* Scale available from the National Center for PTSD at www.ptsd.va.gov; 2013.

12. Kroenke K, Spitzer R, Williams JB. The PHQ-9: Validity of a Brief Depression Severity Measure. *J Gen Intern Med*. 2001;16: 603-13.

13. Spitzer RL, Kroenke K, Williams JB, Löwe B. A brief measure for assessing generalized anxiety disorder: the GAD-7. *Arch Intern Med*. 2006;166(10): 1092-7.

14. Babor TF, Higgins-Biddle JC, Saunders JB, Monteiro MG. *The Alcohol Use Disorders Identification Test, Guidelines for Use in Primary Care. 2nd ed.* Geneva, Switzerland: Department of Mental Health and Substance Dependence, World Health Organization; 2001.

15. Robinson, M., Steen, M., Robertson, S., & Raine, G. (2014). Evaluation of the local Mind resilience programme. *Final report, July*.

16. Eysenck SBG, Eysenck HJ, Barrett P. A revised version of the psychoticism scale. *Pers Indiv Dif*. 1985;6(1): 21-29.

17. Foresight Mental Capital and Wellbeing Project (2008). Final Project report. The Government Office for Science, London.

18. Koivumaa-Honkanen, H., Koskenvuo, M., Honkanen, R. J., Viinamäki, H., Heikkilä, K., & Kaprio, J. (2004). Life dissatisfaction and subsequent work disability in an 11-year follow up. Psychological Medicine 34, 221-228.

19. Lyubomirsky, S., King, L., & Diener, E. (2005). The benefits of frequent positive happiness lead to success? Psychological Bulletin, 131, 803-855.

20. Shepherd, L. & Wild, J. (2013). Cognitive appraisals, objectivity, and coping in ambulance workers: a pilot study. *Emergency Medicine Journal, 31,* 41-4.

21. Kuehl KS, Elliot DL, Goldberg L, Mackinnon DP, Vila BJ, Smith J, Miočević M, O'Rourke HP, Valente MJ, DeFrancesco C, Sleigh A, McGinnis W. The safety and health improvement: enhancing law enforcement departments study: Feasibility and findings. *Frontiers in Public Health*. 2014. 2(38), 1-7.

18. APPENDIX A: Trial Flow Chart

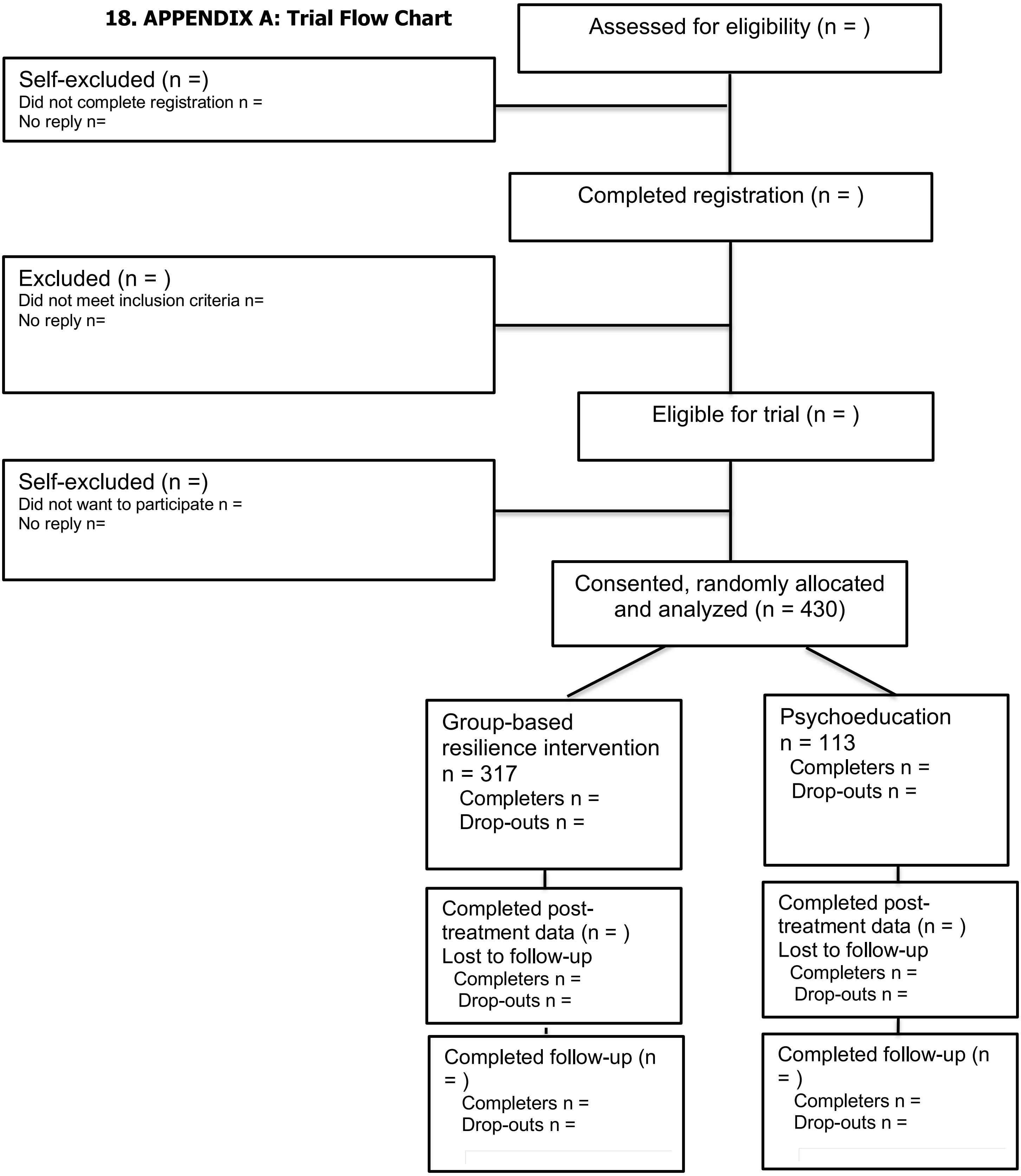

19. APPENDIX B: AMENDMENT HISTORY

Amendment No.	Protocol Version No.	Date issued	Author(s) of changes	Details of Changes made

Protocol amendments will be submitted to the REC and recorded in the Amendment History form.
