# Supplementary material for: Evaluating the effectiveness of a group-based resilience intervention versus psychoeducation for emergency responders in England: A randomised controlled trial
Source: PLoS One. 2020 Nov 12;15(11):e0241704. doi: 10.1371/journal.pone.0241704 (PMC7660584; doi:10.1371/journal.pone.0241704)
Supplement: S3 File — (DOCX) [file pone.0241704.s003.docx]

**S2 Secondary Outcomes**

**Table 5. Secondary Outcome Measures at Baseline, Post-intervention and Follow-up.**

|  | **Resilience Intervention** | | | **Psychoeducation** | | |
| --- | --- | --- | --- | --- | --- | --- |
|  | **Baseline**  **(N=314)** | **Post**  **(N=256)** | **Follow-up**  **(N=281)** | **Baseline**  **(N=113)** | **Post**  **(N=92)** | **Follow-up**  **(N=100)** |
|  | **Mean (SD)** | **Mean (SD)** | **Mean (SD)** | **Mean (SD)** | **Mean (SD)** | **Mean (SD)** |
| Depressive Attributions (DAQ) | 21.90 (8.39) | 20.69 (7.86) | 20.93 (8.87) | 23.05 (8.61) | 22.73 (9.93) | 20.68 (7.79) |
| Coping Behaviour: |  |  |  |  |  |  |
| Adaptive | 15.68 (3.31) | 15.70 (3.38) | 15.69 (3.47) | 16.21 (3.36) | 15.78 (3.42) | 15.87 (3.74) |
| Dysfunctional | 23.21 (6.16) | 22.72 (5.98) | 22.15 (6.40) | 23.70 (6.34) | 22.93 (6.42) | 22.13 (6.30) |
| Response to Intrusive Memories: |  |  |  |  |  |  |
| Suppression | 8.47 (3.61) | 8.85 (3.31) | 8.60 (3.77) | 9.52 (3.86) | 9.04 (3.88) | 8.59 (3.50) |
| Rumination | 7.49 (5.56) | 6.93 (4.99) | 6.71 (5.61) | 7.13 (5.32) | 7.08 (5.63) | 6.58 (5.53) |
| Numbing | 3.33 (2.86) | 3.37 (2.69) | 3.25 (2.96) | 3.35 (2.59) | 3.40 (2.66) | 3.13 (2.36) |
| Rumination (RRS) | 38.77 (12.90) | 37.49 (12.20) | 37.03 (13.66) | 39.23 (13.38) | 38.73 (14.04) | 36.86 (13.73) |
| Trauma Exposure | 4.65 (3.93) | 3.97 (4.06) | 3.50 (3.84) | 4.99 (4.27) | 4.91 (3.79) | 4.34 (4.38) |
| Median (IQR): | 4 (4.00) | 3 (5.00) | 3 (4.0) | 5 (5.00) | 5 (6.00) | 4 (5.00) |
| PTSD (PCL-5) | 8.98 (12.41) | 7.49 (10.46) | 7.38 (11.61) | 9.42 (13.75) | 9.11 (13.56) | 8.68 (14.55) |
| Median (IQR): | 3.0 (13.00) | 4.0 (10.00) | 2.0 (9.00) | 4.0 (12.50) | 3.5 (10.00) | 2.0 (11.25) |
| Depression (PHQ-9) | 3.89 (4.07) | 3.49 (3.19) | 3.38 (3.81) | 3.83 (3.85) | 3.82 (4.42) | 3.39 (4.05) |
| Median (IQR): | 3 (4.00) | 3 (5.00) | 2 (5.00) | 3 (5.00) | 3 (5.00) | 2 (5.25) |
| Anxiety (GAD-7) | 3.22 (3.61) | 3.17 (3.12) | 3.00 (3.44) | 3.35 (3.63) | 3.27 (3.43) | 2.98 (3.71) |
| Median (IQR): | 2 (5.00) | 2 (3.00) | 2 (4.00) | 3 (5.00) | 2 (5.00) | 2 (4.00) |
| Alcohol Use (AUDIT) | 5.23 (4.08) | 4.82 (3.70) | 4.83 (4.07) | 5.19 (4.25) | 5.20 (4.54) | 4.95 (4.02) |
| Neuroticism (EPQ) | 5.43 (3.42) | 5.20 (3.36) | 5.06 (3.52) | 4.95 (3.46) | 4.88 (3.51) | 4.85 (3.28) |

*Note.* Median and IQR provided where SD is larger than the mean. DAQ = Depressive Attributions Questionnaire; RRS = Ruminative Responses Scale; PCL= Posttraumatic Stress Disorder Checklist; PHQ = Patient Health Questionnaire; GAD = Generalised Anxiety Disorder questionnaire; AUDIT = Alcohol Use Disorders Identification Test; EPQ = Eysenck Personality Questionnaire.

Table 6

*Linear regression models to examine baseline neuroticism scores as a predictor of change in the primary outcome measures among the intervention group.*

| Dependent Variable | Overall model | | |  | Neuroticism | |
| --- | --- | --- | --- | --- | --- | --- |
|  | *F*(4, 250) | *p* | *R*^2^ |  | *beta* | *p* |
| WEMWS | 0.923 | .451 | 0.015 |  | −0.207 | .188 |
| CDRISC | 1.130 | .341 | 0.018 |  | −0.282 | .255 |
| GSES | 2.270 | .062 | 0.035 |  | −0.199 | .003 |
| SPS | 0.984 | .417 | 0.016 |  | −0.261 | .314 |
| SS (home) | 1.560 | .186 | 0.024 |  | −0.120 | .208 |
| SS (work) | 0.304 | .875 | 0.005 |  | −0.059 | .475 |

*Note.* Dependent variables used residualised gain scores. Baseline score, Gender, and Site included as covariates in each model. WEMWBS = Warwick Edinburgh Mental Wellbeing Scale; CDRISC = Connor-Davidson Resilience Scale; GSES = General Self-Efficacy Scale; SPS = Social Participation Scale; SS = Social Support.
