# Supplementary material for: Evaluating the effectiveness of a group-based resilience intervention versus psychoeducation for emergency responders in England: A randomised controlled trial
Source: PLoS One. 2020 Nov 12;15(11):e0241704. doi: 10.1371/journal.pone.0241704 (PMC7660584; doi:10.1371/journal.pone.0241704)
Supplement: Attachment — Submitted filename: Response to Reviewers.docx [file pone.0241704.s005.docx]

Dr Jennifer Wild, [jennifer.wild@psy.ox.ac.uk](mailto:jennifer.wild@psy.ox.ac.uk)
Department of Experimental Psychology
University of Oxford, OX1 1TW

Dr Graham Thew, [graham.thew@psy.ox.ac.uk](mailto:graham.thew@psy.ox.ac.uk)
Department of Experimental Psychology

University of Oxford, OX1 1TW

9 October, 2020

Yutaka J. Matsuoka, MD, PhD
Academic Editor
PLOS ONE

Dear Professor Matsuoka

Re: Evaluating the effectiveness of a group-based resilience intervention versus psychoeducation for emergency responders in England: A randomised controlled trial

Thank you for reviewing the above paper. We are grateful for you and the reviewers’ careful readings of the paper. We have now incorporated the helpful suggestions of Reviewer 2. We believe the presentation of the manuscript has been further strengthened and trust that it is now acceptable for publication.

For clarity, each of the revisions is outlined below. Changes to the manuscript are shown in quotations below.

Review Comments to the Author

Reviewer #2: I would point out that authors made significant improvements in the paper. Now it is much clearer what are analyses referring too and how they can be interpreted, at least to me. However I still have few minor concerns:

1. “The Time*Condition interaction is therefore not estimated explicitly (as in ANCOVA)...” – I would suggest that authors mention this somewhere in the text or so. Maybe I was not reading carefully but since it confused me, I believe it will confuse many readers of the paper.

Thank you for this suggestion. We have added the below sentence into the Method in order to clarify that the Time*Condition interaction enables the calculation of the treatment effect at each timepoint. We hope that this makes it clearer for readers that the treatment effect at each timepoint is therefore the main outcome of interest.

“The time-by-condition interaction was also entered as a fixed effect in order to allow the estimation of the treatment effect at each timepoint.”

2. I think it would be useful to mention somewhere in analysis part or maybe in the results part, that effect size measures in some sense represent the other approach to group difference testing. The first one is by Linear Mixed-Effects Models, and the other one (more similar to classical ones) is effect sizes approach (targeting mean differences instead of residuals). I strongly believe that this would help in clarity of the text for many readers.

We have amended the below sentence from the Method to incorporate your suggestion, and provide a clearer rationale for the additional use of Effect sizes.

“Between-group effect sizes (*d*_Cohen_), based on a mean differences approach, were then calculated from the results of these models in order to assist with interpretation of the findings.”

3. I would suggest showing results for the secondary measures, too, at least in the appendix. It would be easier to see, track and so on. For instance, primary measures effect sizes are shown in table, but vaguely commented, while secondary measures differences are commented in more details, and no table is shown in the text. I think that adding it at least in appendix would make it a bit clearer, since readers might see the data text is referring too.

>>Thank you for this suggestion. All secondary outcomes are shown in Tables 5 and 6 in the Supporting Information files. These were submitted in the previous revision and can be found on the page of the submission that says “Click here to download Supporting Information S2 Secondary Outcome Table 5” and “Click here to download Supporting Information S2 Secondary Outcome Table 6.”

4. Authors write “...receiving psychoeducation completed a mean number of 328 4.71 (SD=2.01) modules, which was significantly greater than the number of sessions 329 attended (F(1,429)=7.21, p=0.008)”, but it confuse me, since I do not understand how can a number of completed be greater than the number of attended sessions? Maybe I misunderstood it, but I believe that other readers can misunderstand it too, so at least it requires come kind of explanation.

>>We agree it would be helpful to clarify this further in the text. The modules formed the psychoeducation condition and group sessions formed the resilience intervention. We have clarified this point on lines 326 to 329:

Participants receiving the resilience intervention completed a mean number of 4.11 group sessions (SD=2.02) whilst those receiving the psychoeducation intervention completed a mean number of 4.71 (SD=2.01) modules. Participants receiving psychoeducation completed more modules than sessions attended by participants in the resilience intervention (F(1,429)=7.21, p=0.008).

Thank you again for your time and attention. We look forward to hearing from you.

Yours sincerely,

Dr Jennifer Wild & Dr Graham Thew
